# Can Vitamin D Therapy Contribute to the Conservative Resolution of Osteolytic Lesions of the Jaws?

**DOI:** 10.1155/2021/5510724

**Published:** 2021-07-17

**Authors:** Kamis Gaballah, Sami Kenz, Raeefa Anis, Omar Kujan

**Affiliations:** ^1^Department of Oral and Craniofacial Health Sciences, College of Dental Medicine, University of Sharjah, UAE; ^2^Rashid Centre for Diabetes & Research, Ajman, UAE; ^3^Ajman University, UAE; ^4^UWA Dental School, The University of Western Australia, Perth WA6009, Australia

## Abstract

Osteolytic lesions of the jaw are not uncommon. Such lesions usually arise from local pathologies, but some have systemic backgrounds. We describe a 12-year-old girl who presented with an asymptomatic left mandibular swelling. The bony swelling was corresponding to a radiolucent lesion in the left premolar/molar region. This lesion could have represented an inflammatory and developmental odontogenic jaw cyst, giant cell lesion, and odontogenic tumor. However, the workup investigations revealed secondary hyperparathyroidism due to vitamin D deficiency. A vitamin D replacement was initiated with a single I.M. injection of 300,000 I.U followed by 10,000 I.U orally, weekly. Six weeks later, her Vitamin D and parathyroid hormone were normalized, and she showed significant clinical and radiological improvement of the jaw lesion. At 18 months, follow-up the panoramic image revealed complete resolution of the radiolucency and stable normal parathyroid hormone and vitamin D levels. In conclusion, Jaw bone lesions can develop secondary to hyperparathyroidism due to vitamin D deficiency, and this should be ruled out before any surgical intervention. Treatment of such lesions lies in the correction of parathyroid excess with a careful and systematic approach. This may prevent unnecessary surgical intervention in such patients.

## 1. Introduction

Vitamin D deficiency is a health burden that has caused concerns worldwide over the past decades due to implications on bone biology, multiple sclerosis, diabetes, depression, and cardiovascular disease [1]. Despite the sunny climate, a prevalence of 90.9% of hypovitaminosis D was reported in women in the UAE [2, 3]. Likewise, Yammine et al. reported that 57.5% of the UAE general population were vitamin D deficient, and 27.8% had vitamin D levels between 21 and 30 ng/ml, denoting insufficiency below the normal average [4]. Vitamin D deficiency leads to decreased serum calcium levels, resulting in elevated parathyroid hormone (PTH) secretion; therefore, calcium from the bones, kidneys, and intestines will be released. In effect, chronic vitamin D deficiency leads to secondary hyperparathyroidism [5].

Radiolucent lesions of the jaws are not uncommon, often detected in conventional radiography, and could be odontogenic or non-odontogenic [6]. The process of obtaining a definitive diagnosis and formulating a treatment plan is initiated by performing a surgical biopsy of the concerning lesion. However, the exclusion of bone homeostasis abnormalities, such as primary or secondary hyperparathyroidism, should be considered in many cases [6]. Management of the jaw's osteolytic lesions, depending on the diagnosis, ranges from minimal intervention by intralesional corticosteroids, calcitonin therapy, and cryotherapy to surgical intervention followed by jaw reconstruction [7]. We present a rare case of an osteolytic lesion associated with secondary hyperparathyroidism due to vitamin D deficiency, treated with vitamin D supplementation. Our case presentation's findings suggest an altered approach to diagnosing and managing osteolytic lesions before surgical exploration.

## 2. Case Presentation

**Figure 1:**
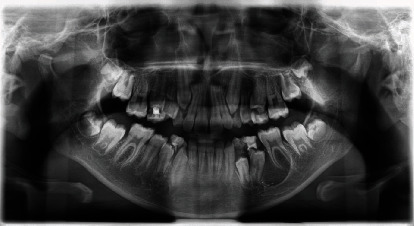
Pre-Op Orthopantomograph showing a well-defined radiolucent lesion in the left posterior mandible.

A 12-year-old female orthodontist was referred to Dr. Gaballah to assess and treat the radiolucent lesion in the mandibular left premolar-molar area. The lesion was identified during routine workups for orthodontic treatment to address anterior teeth' crowding (Figure 1). Clinically, the patient had an asymptomatic buccal extension of the cortical plate. The lump was roughly 18x22 mm and was not associated with any pocketing of erupted teeth or sinus or cervical lymphadenopathy. There were no neurosensory deficits; the teeth involved were caries-free and not mobile. The case's medical history was unremarkable, although the patient complained of lethargy and general fatigue as defined by the mother.

**Figure 2:**
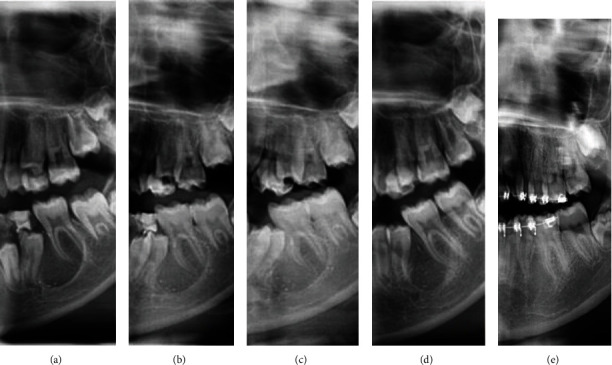
shows panoramic radiographs taken at initial and subsequent follow-up visits (a). The preoperative imaging referred by the orthodontist. (b) The lesion one-month post-intervention (c) At 3-month follow-up post-intervention. (d) At nine-month follow-up. (e) At 18 months of follow-up post-intervention showing complete resolution.

Panoramic imaging showed a well-defined unilocular ovoid radiolucent lesion with a sclerotic rim extending from the distal aspect of the mandibular second premolar to the distal surface of the distal root of the mandibular first molar (Figure 1 and 2(a)). There was no sign of root resorption. A preliminary differential diagnosis, which included inflammatory and developmental odontogenic jaw cysts, giant cell lesions, and odontogenic tumors, was made because of the clinical and radiological findings. A comprehensive blood test was performed along with the measurement of parathyroid hormone (PTH) and renal function tests (RFT). Laboratory findings were within the range, but PTH was high at 81.3 (15-68) pg/mL and low normal serum calcium at 2.17 mmol/L (2.15-2.6), and low vitamin D 32 (75-200) nmol/L. The primary hyperparathyroidism was excluded with the use of USS thyroid. Thus, the patient was diagnosed with secondary hyperparathyroidism due to vitamin D deficiency and associated with a left mandible lytic lesion.

Vitamin D deficiency was treated as indicated by the endocrinologist before any surgery to correct hyperparathyroidism. As a result, the surgical biopsy was postponed. Treatment was begun correcting the hypovitaminosis with a single injection of Vitamin D3 300,000 I.U complemented by 10,000 I.U orally once a week. Six weeks post-intervention, a repeat vitamin D test showed a vitamin D level of 85 nmol/L with PTH lowered to 38 and serum calcium 2.4 mmol/L suggesting a substantial improvement in parathyroid function and calcium homeostasis. Parents also observed an increase in the patient's physical activity and cognitive performance. The same visit's panoramic follow-up showed some signs of bone formation in the affected region (Figure 2(b)).

The patient was kept on a maintenance dose of 10000 D3 I.U weekly. Based on progress in clinical, laboratory testing, and imaging of the jaw lesions (Figure 2(c)), the parents declined any surgical intervention, including biopsy, and agreed to pursue periodic follow-up. A Radiograph was taken at nine months, followed by further progress with new bone formation (Figure 2(d)). 4Repeat laboratory analysis also showed regular intact PTH 49.3 (15-68) pg./ml, vitamin D (25(OH)D) 73.3 (30-100) ng/ml and calcium 2.5 (2.15-2.60) mmol/L. The patient proceeded with 10,000 D3 I.U weekly and followed up with 18-month post-medical action. The last Panoramic picture showed a full resolution of the lesion (Figure 2(e)), and the laboratory tests confirmed stable values of PTH (31.5 pg/ml), vitamin D (53.53 ng/ml), calcium 2.5 (2.15-2.60) mmol/L. CBC and RFT within the reference scales. Following complete lesion resolution and bone homeostatic parameters correction, the patient was referred back to her orthodontist to resume the treatment.  Table 1 summarizes the biochemistry panel of the patient before, after the medical intervention, and eighteen-month follow-up values.

## 3. Discussion

Despite the favorable latitude and abundance of sunlight in the Gulf region, the UAE population remains vitamin D deficient. Due to excessive heat and humidity in the relatively long summer season, increased sun avoidance, particularly in females, is noticed among the inhabitants [8–10]. Contrary to findings in other regions, increased sun exposure in the UAE occurs during winter when the temperatures are comparatively lower. Females in the UAE and the Middle East may have considerably less sunlight exposure due to their dressing styles covering most or all of the body for cultural or religious reasons [8–10]. Adolescents are at higher risk of vitamin D deficiency due to current trends and preference for indoor activities, computers, mobiles, social media, gaming, and watching television [9]. Vitamin D deficiency results in reduced intestinal calcium absorption and a lower ionized calcium level. This leads to increased secretion of the PTH. The hormone promotes the reabsorption of calcium from renal tubules. It also causes the increased conversion of calcidiol to calcitriol, which leads to increased intestinal calcium absorption and enhanced mobilization of calcium from bone [9]. The secondary hyperparathyroidism that occurs, as a result, maintains serum calcium in the normal range at the expense of mobilization of calcium from the skeleton and an increase in phosphorous wasting in the kidneys [11]. This PTH-mediated rise in osteoclastic activity causes an overall reduction in bone mineral density [11]. In our case, vitamin D deficiency contributed to excessive secretion of PTH, which resulted in an imbalance in osteoclastic-osteoblastic homeostasis, increased mobilization of calcium from bone, and ultimately presented as an osteolytic lesion in the mandible.

Given the clinical and laboratory findings, the biopsy was not performed. We are conscious of the limitation that we were unable to characterize the lesion histopathologically. However, the significant clinical and radiological resolution of the lesion and correction of the secondary hyperparathyroidism bears evidence to the preliminary diagnosis of a brown tumor of secondary hyperparathyroidism caused by vitamin D deficiency. The biopsy was considered on the initial surgical plan. However, discovering the abnormal bone homeostasis and the improvement noted at the early stage of the Vitamin D replacement make the family deny that option. Keeping in mind, potential complications of surgical biopsy may include damage to surrounding tissue, excessive bleeding, bone fractures, and infection. However, in most cases, biopsy benefit generally overrides the risks in diagnosis and treatment planning. Brown tumor, also known as osteitis fibrosa cystica, is a benign, non-neoplastic, giant cell lesion that arises from excessive PTH levels due to primary or secondary hyperparathyroidism. They are preferentially found in the long bone, ribs, clavicle, pelvic girdle, and mandible. However, They can occur in any skeletal region in the body [12].

In contrast, this manuscript is written a report documented a potential cause of a brown tumor of the left proximal femur in a 16-year-old female treated with calcium vitamin D supplementation [12]. Complete regression of the lesion was noted, and a diagnosis of the brown tumor was considered in the absence of histopathological evaluation due to the treatment's success. Similar to our case report, the authors also advocate biochemical investigations of an osteolytic lesion to rule out brown tumor of hyperparathyroidism before any surgical intervention. This paper sheds light on a potential conservative approach for managing osteolytic lesions associated with hyperparathyroidism reducing morbidity incurred by surgical management, which is commonly practiced.

## Figures and Tables

**Table 1:** the full biochemistry panel of the patient before, after the medical intervention, and eighteen-month follow-up values.

	Range value	At presentation	Six weeks post-medical intervention	Nine months post-medical intervention	Eighteen months post-medical intervention
PTH	(15-68) pg/mL	81.3	38	49.3	31.5
Serum calcium	(2.15-2.6) mmol/L	2.17	2.4	2.4	2.5
Vitamin D 32	(75-200) nmol/L	32	85	73.3	63.53
Creatinine	49-90 umol/l	50	53	51	54
Urea	2.5-6 mmol/l	4.3	4.3	4.2	4.3
Total CO_2_	20-29 mmol/l	23	23	25	23
Sodium	136-145 mmol/l	139	140	140	139
Potassium	3.5-5.1 mmol/l	4.2	4.2	4.1	4.1
Chloride	98-107 mmol/l	101	102	102	101
Calcium	2.15-2.8 mmol/l	2.4	2.4	2.3	2.3
Alkaline phosphatase	28-300 u/l	345	303	191	78

## Data Availability

Data are available on request.

## Abbreviations

PTH: ParaThyroid hormone
RFT: Renal function tests
25(OH)D: 25-hydroxyvitamin D
UAE: United Arab Emirates
CBC: Complete blood count.
